# A talkative Potts attractor neural network welcomes BLISS words

**DOI:** 10.1186/1471-2202-13-S1-P21

**Published:** 2012-07-16

**Authors:** Sahar Pirmoradian, Alessandro Treves

**Affiliations:** 1 Cognitive Neuroscience Sector, SISSA, Trieste, 34136, Italy

## 

Neuroscientists have observed that the human brain is comprised of neurons. We have observed that babies start speaking at an early age, yet no young animals, including pets, have so far been seen to speak, at least not in the articulated fashion of human babies. To understand this highly cognitive ability, many psycholinguistic data have been gathered, from behavioral, to neurolinguistic, to recent neuroimaging studies, each measuring macroscopic properties of the brain. Nevertheless, the challenging question remains unanswered of how such complicated behavior emerges from the microscopic (or mescoscopic) properties of individual neurons and of networks of neurons in the brain.

We would like to tackle this question by developing and analyzing a Potts attractor neural network model, whose units hypothetically represent patches of the cortex. The network has the ability to spontaneously hop (or latch) across memory patterns (which have been stored as dynamical attractors), thus producing an infinite sequence of patterns, at least in some regimes [1]. We would like to train the network with a corpus of sentences in BLISS [2]. BLISS is a scaled-down synthetic language of intermediate complexity, with about 150 words and about 40 rewrite rules. We expect the Potts network to generate sequences of memorized words, with statistics reflecting to some degree that of the BLISS corpus used in training it.

Before training the network on the corpus, the critical issues to be addressed, and the central ones here, are: how should the words be represented in a cognitively plausible manner in the network? how should the correlation between words, in terms of both meaning and statistical dependences, be reflected in their (neural) representations? how should two main characteristics of a word, the meaning (semantic) and the syntactic properties, be represented in the network?

We represent words in a distributed fashion on 900 units, 541 out of which express the semantic content and the rest, 359 units, are representative of the syntactic characteristics of a word. The distinction between the semantic and syntactic characteristics of a word has been loosely inspired by a vast number of neuropsychological studies [3]. Further, several findings have indicated a distinction between the encoding of function words (i.e. prepositions, conjunctives, determiners, etc.) and content words (i.e. nouns, verbs, adjectives, ...) in the brain [4]. To implement a plausible model of the variable degree of correlation between word representations, we have used an algorithm comprised of two steps [5]: first, a number of vectors, called factors, are established, each factor influencing the activation of some of the units, by "suggesting" a particular state; second, the competition among these factors determines the activation state of each unit of a word.

The preliminary analysis of the produced patterns indicates the resemblance between the statistics of the representation of words and the patterns that can generate the latching behavior of the network. This is a promising step towards building a neural network that can spontaneously generate a sequence of words (sentences) with desired syntactic and semantic relationships between words in sentences.

## References

[B1] Russo Eleonora Pirmoradian Sahar Treves Alessandro Associative Latching Dynamics vs. Syntax Adv in Cogn Neurodyn (II) 2011 Springer 111 115

[B2] Pirmoradian Sahar Treves Alessandro BLISS: an artificial language for learnability studies Cogn Comput 2011 3 539 553 10.1007/s12559-011-9113-4

[B3] Shallice Tim Cooper Richard The organisation of mind 2011 Oxford Univ Pr 10.1016/j.cortex.2011.07.004 23040241

[B4] Shapiro Kevin A Caramazza Alfonso Morphological Processes in Language Production The Cognitive Neurosciences 2011 MIT Pr 777 788

[B5] Treves Alessandro Frontal latching networks: a possible neural basis for infinite recursion Cogn Neuropsych 2005 3 276 291 10.1080/02643290442000329 21038250

